# A Review on Ionic Liquids-Based Membranes for Middle and High Temperature Polymer Electrolyte Membrane Fuel Cells (PEM FCs)

**DOI:** 10.3390/ijms22115430

**Published:** 2021-05-21

**Authors:** Mohammad Ebrahimi, Wojciech Kujawski, Kateryna Fatyeyeva, Joanna Kujawa

**Affiliations:** 1 Faculty of Chemistry, Nicolaus Copernicus University, 7 Gagarina Street, 87-100 Toruń, Poland; mohammad.ebrahimi@doktorant.umk.pl (M.E.); joanna.kujawa@umk.pl (J.K.); 2 Normandie Univ, UNIROUEN, INSA ROUEN, CNRS, Polymères Biopolymères Surfaces (PBS), 76000 Rouen, France; kateryna.fatyeyeva@univ-rouen.fr

**Keywords:** middle and high temperature polymer electrolyte membrane fuel cells, polymer electrolyte membranes, ionic liquids, proton conductivity, leaching

## Abstract

Today, the use of polymer electrolyte membranes (PEMs) possessing ionic liquids (ILs) in middle and high temperature polymer electrolyte membrane fuel cells (MT-PEMFCs and HT-PEMFCs) have been increased. ILs are the organic salts, and they are typically liquid at the temperature lower than 100 °C with high conductivity and thermal stability. The membranes containing ILs can conduct protons through the PEMs at elevated temperatures (more than 80 °C), unlike the Nafion-based membranes. A wide range of ILs have been identified, including chiral ILs, bio-ILs, basic ILs, energetic ILs, metallic ILs, and neutral ILs, that, from among them, functionalized ionic liquids (FILs) include a lot of ion exchange groups in their structure that improve and accelerate proton conduction through the polymeric membrane. In spite of positive features of using ILs, the leaching of ILs from the membranes during the operation of fuel cell is the main downside of these organic salts, which leads to reducing the performance of the membranes; however, there are some ways to diminish leaching from the membranes. The aim of this review is to provide an overview of these issues by evaluating key studies that have been undertaken in the last years in order to present objective and comprehensive updated information that presents the progress that has been made in this field. Significant information regarding the utilization of ILs in MT-PEMFCs and HT-PEMFCs, ILs structure, properties, and synthesis is given. Moreover, leaching of ILs as a challenging demerit and the possible methods to tackle this problem are approached in this paper. The present review will be of interest to chemists, electrochemists, environmentalists, and any other researchers working on sustainable energy production field.

## 1. Introduction

**Figure 1:**
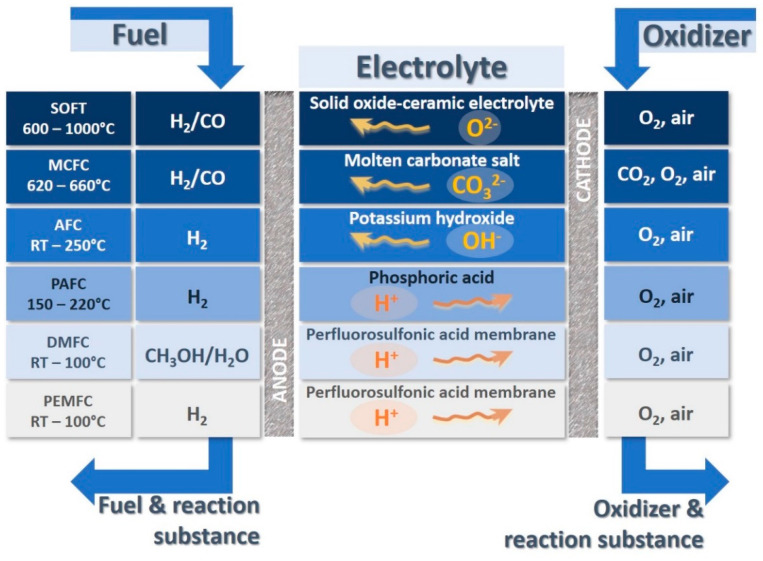
Overview of the various types of fuel cells with the following operation temperatures: polymer electrolyte membrane fuel cell (PEMFC) and direct methanol fuel cell (DMFC) RT–100 °C, phosphoric acid fuel cell (PAFC) 150–220 °C, alkaline fuel cell (AFC) RT–250 °C, molten carbonate fuel cell (MCFC) 620–660 °C, and solid oxide fuel cell (SOFC) 600–1000 °C. RT = room temperature.

**Figure 2:**
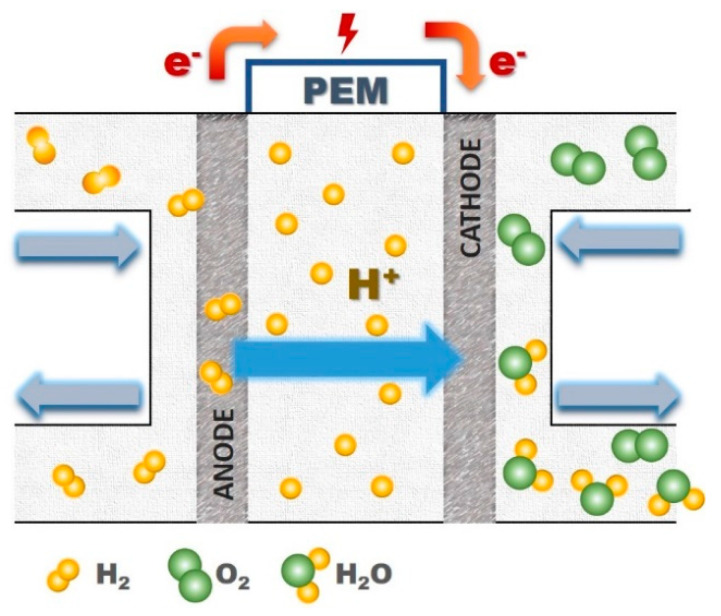
The general schematic of PEMFC. Reprinted with permission from Reference [23]. Copyright 2020 De Gruyter.

Today, individuals have a special interest in applying renewable sources of energy, such as solar, wind, biogas, geothermal, biomass, low-impact hydroelectricity, emerging technologies-wave, tidal power, and fuel cells [1,2,3,4,5]. From among them fuel cells have been widely used [5,6,7,8]. In fact, there are a growing number of reasons behind the widespread use of this sort of device [5,6,7,8,9]. The most crucial reason is that fuel cell is considered as an eco-friendly process which does not harm to the nature, and it does not produce any contamination [5,10]. In contrast, nonrenewable sources of energy (generally combustion of fossil fuels) cause serious environmental issues, including global warming, air pollution, acid rains, and ozone depletion [10,11,12,13]. Furthermore, they can also result in several major health problems, like cancer, cardiovascular disease, and respiratory problems [14,15,16,17]. Consequently, humans should care about their future and next generation since the resources of the earth (especially energy) are limited and irreversible [18,19]. Fuel cells can be a great environmentally friendly alternative instead of fossil fuels which are able to convert chemical energy into electrical one [20,21,22]. A wide range of fuel cells have been utilized depending on the kinds of electrolytes, operational conditions (temperature and humidification), as well as technological and design factors, such as polymer electrolyte membrane fuel cell (PEMFC), solid oxide fuel cell (SOFC), molten carbonate fuel cell (MCFC), alkaline fuel cell (AFC), direct methanol fuel cell (DMFC), and phosphoric acid fuel cell (PAFC) (Figure 1) [22,23]. These days, research findings confirm that PEMFC can have a good potential for using in lab, semi-industrial, and even industrial scales [23,24]. Owing to PEMFC high effectiveness and efficiency, favorable power density, and quick startup, it has been noticeably employed in various subjects, including transportation, portable gadgets, and stationary power production [25,26]. Besides, in each PEMFC, hydrogen and oxygen (or air) generally react with one another as fuel and oxidizer agents, respectively, and, finally, the products of this reaction are heat, water, and electricity [10,27]. It is clear that this type of electrochemical device would be a very promising candidate for sustainable energy production. PEM is the most important part of each PEMFC [23,26,28]. Actually, PEM plays an essential role in transporting protons through the ion exchange membrane from anodic section to cathodic one, and its proper performance in this matter can be a decisive factor in PEMFC efficiency (Figure 2) [29,30,31].

In fact, proton conduction through the membrane according to the operating conditions can be different [32,33]. Usually, the vehicle and Grotthuss mechanisms are valid and accepted to express proton conducting among scientists [34,35]. These two mechanisms are defined depending on two decisive operating factors: humidity and temperature [35,36]. Generally, the vehicle mechanism is employed at low temperature and high moisture conditions [37]. In this pattern, water plays a vital role in transporting protons from one side to another side of membrane [38]. Due to the fact that water carries the protons through the membrane (like an automobile), this mechanism is known as a vehicle [39]. For instance, proton transportation within Nafion membrane is carried out by this mechanism [40]. Water and sulfonate (SO_3_^−^) groups provide the suitable condition for conducting hydrogen ions across the Nafion membrane [40]. On the other hand, at elevated temperature and low humidification, using Grotthuss mechanism is valid, which is also recognized as proton hopping mechanism [41]. Here, water cannot act as ion transporter since the temperature is too high, and water will be evaporated [42]. Hence, in this pattern, protons are moved via forming and deforming hydrogen bonds [43]. In general, every PEMFC has the same body structure, which is called membrane electrode assembly (MEA) [44]. PEM, anode, and cathode catalyst layers, anode and cathode gas diffusion layers, and gaskets are placed in MEA, and each part has its own responsibility [23]. Since PEMFC has some negative features, it has been unable to be used on industrial scale [45]. It can be mentioned that these PEMFC demerits are related to the too much expense of PEM (Nafion is a case in point) and catalyst, facing serious problems in the case of water management in the moisture and aqueous-free conditions [23,45]. A wide range of polymers have been used for this goal, but, thanks to the tunable properties of Nafion (the most common types of perfluorosulfonic acid (PSFA) ionomers), it is widely-used [46,47,48]. Research findings confirm that there are a lot of benefits to the usage of Nafion membranes at lower temperatures and under hydrous situations, such as great proton conductivity and excellent mechanical, thermal, chemical, and oxidative stability [49]. However, this special polymeric membrane has several drawbacks, of which the most impractical character would be that Nafion can only be used conveniently at low temperatures, and, in order to conduct protons through this sort of polymer, the presence of water is vital [49,50]. Actually, in the range of water boiling temperature, the quick dehydration is unavoidable, which can have several adverse impacts upon fuel cell performance, including diminishing conductivity, and, in some instances, it leads to immutable variations in the microstructure of membrane [51]. There might be a number of reasons behind the considerable reduction of the proton conductivity of the Nafion membrane [49,50]. Nonetheless, it is believed that the most sensible cause for this phenomenon is because of the evaporation of water from Nafion membrane [51,52]. The range of operation temperature for Nafion membrane is too limited, and it has a positive efficiency just between 60 and 80 °C, but higher temperatures are desirable due to the several reasons [23]. Researchers argue that the one that really stands out is that the exploitation of PEMFC at elevated temperatures can bring about accelerating the electrochemical reactions at anode and cathode (quicker electrode kinetics) [53]. Moreover, another reason is that utilization of PEMFC in this condition can be really profitable as it is possible to use hydrogen as fuel with lower purity [53]. Additionally, using the other fuels, such as biogas, natural gas, and methanol, are feasible [10,53]. Furthermore, the application of PEMFC at more than 80 °C could reduce the carbon monoxide (CO) poisoning at electrodes which it is one of the most challenging problems for low temperature polymer electrolyte membrane fuel cells (LT-PEMFCs) [53,54,55]. It also can result in facilitating heat and water management [10]. A wide range of investigations have been conducted by researchers for finding new other electrolyte materials that can be used at middle and high temperatures and under aqueous-free conditions to retain suitable proton conductivity, thermal stability, and mechanical resistance [23,53,56,57]. Dehydration in PEMFC is the biggest problem here because all of these materials can work under fully moisture condition [23]. A lot of efforts have been done so far to improve this important problem, such as incorporation of inorganic and organic nanoparticles (NPs), nanosheets, nanotubes, and fillers, e.g., ZrO_2_, TiO_2_, SiO_2_, P_2_O_5_, Fe_2_TiO_5_, BaZrO_3_, Zeolite NPs, aluminosilicate, graphene oxide (GO), zirconium phosphate, titanium dioxide nanotube, nanowire, and heteropolyacid, as well as the use of sulfonated hydrocarbon polymers, e.g., sulfonated poly(ether ether) ketone (SPEEK) and sulfonated polyimide (SPI) [53,58,59,60,61,62,63,64,65,66,67,68,69]. Even though these enhancements could result in some advantages about obtaining MT-PEMFC and HT-PEMFC, they require water in order to pass protons through the PEM. Thus, these ways cannot be regarded as successful method to gain HT-PEMFC [70]. The alteration of water with none or less volatile solvents is an innovative way to exploit PEM at middle and elevated temperatures [23,44]. Utilization of phosphoric acid (PA) can be a good alternative instead of using water as it is a low volatile solvent and independent from humidity [44]. Moreover, the use of PA brings about enhancing proton conductivity remarkably [44,71]. However, it has demerits, such as acid leaching and degradation of membrane (decreasing mechanical stability) [71]. ILs can be good candidates as additives into the PEM in order to use at middle and elevated temperatures without humidification [72]. The focus of this paper is to provide such a review, presented in a systematic and comprehensive way so that the current knowledge on ILs-based membranes and details on their potential for MT-PEMFCs and HT-PEMFCs are provided in one useful source for guiding future studies.

## 2. Ionic Liquids Structure and Properties

Over the last decade, ILs have attracted great attention from scientists and organizations which is sensible by increasing the number of published papers and books [73,74,75]. ILs are organic salts which are made-up by ions (organic cation and inorganic/organic anions) and they are usually liquid at temperature lower than 100 °C [76,77,78,79]. Initially, the word of IL was used and had the same meaning as “molten salt” [80]. However, today, the original meaning is changed and ILs refers to organic compounds which have melting temperature below 100 °C [78,79,80]. The reason why these organic materials are often liquid is owing to the difference between the size of anions and cations [81]. Consequently, the physicochemical bonds and interactions among them are not often strong [81]. In fact, the most prominent features of ILs are negligible vapor pressure [82], high viscosity [83], wide electrochemical window [84], low melting point [85], non-flammability [78,79], great proton conductivity [86], high thermal stability [87], and good chemical stability [88]. Thus, considering these properties, ILs are desirable alternatives for volatile solvents which have many adverse effects upon the nature [89]. Despite the positive features of ILs, the toxicity aspects of these organic salts should be taken into account as an important issue [90]. A wide range of research have been conducted so far concerning ILs toxicity and the results demonstrated that the usage of ILs (e.g., imidazolium and pyridinium-based ILs) even at low values can have several adverse effects upon human [91], animals [92], bacteria [93], and algae [94]. ILs are used for several applications, such as gas separation [95], metal extraction [96], drug delivery [97], and wastewater treatment [98], which can result in dispersion of ILs to the ecosystem. For instance, hydrophobic ILs are utilized for wastewater treatment and they have a good solubility in water (between 10^2^ and 10^4^ mg/L) [90,99,100]. Therefore, these toxic compounds disperse into the water reservoirs, and then soils, plants, animals, and people are affected [101,102]. Up to now, there is a number of efforts which have been done to decrease the toxicity and negative effects of ILs on the environment [90,103]. All these aspects were very well debated in a critical review related to the toxicity of ILs [104]. There is a vast number of ILs and several types of them are displayed in Table S1 considering their full and abbreviated names.

### 2.1. Types of ILs

Today, numerous types of ILs have been known and used in different backgrounds, including task specific-ionic liquids (TS-ILs), room temperature-ionic liquids (RT-ILs), chiral-ionic liquids (C-ILs), switchable polarity solvent-ILs (SPS-ILs), bio-ionic liquids (Bio-ILs), supported ionic liquids (S-ILs), basic ionic liquids (B-ILs), energetic-ionic liquids (E-ILs), neutral-ionic liquids (*N*-ILs), metallic-ionic liquids (M-ILs), poly-ionic liquids (P-ILs), and protic-ionic liquids (Pr-ILs) [78,79,89,105,106,107,108,109,110,111,112,113,114,115,116,117,118,119,120,121]. Indeed, such huge variety of ILs is mainly owing to very high diversity in the number of anions and cations and also their own unique thermal and physicochemical features, including ionic conductivity and the ability to mix in organic and inorganic solvents [78,79]. TS-ILs are one of the most popular ILs as a result of their substantial characteristics [79]. These sorts of ILs can have innumerable functional groups in their structure, for this reason they are also recognized as functionalized ILs [78,79]. However, since these kinds of ILs contain different reactive functional groups, synthesis of them is hard and require much more time compared with other types [78]. Table 1 provides some information about the rest of mentioned ILs.

### 2.2. ILs Synthesis

Depending on the kinds of ILs and user’s requirements, there are assorted techniques for synthesis of ILs; however, these organic compounds are generally synthesized by two main methods [78,89]. Research findings confirm that there are two types of ILs: primary and secondary ILs [89]. The primary ILs are produced by protonation or alkylation of a base by an acid or haloalkane [89,122]. There are four routes to synthesize secondary ILs, including the use of metal salt, Brønsted acid, ion exchange resin, or Lewis acid [78,89,122,123]. In this review the synthesis of ILs by acid-base neutralization, metathesis, and alkylation reactions are going to be presented as these methods are the most common ways for synthesis of primary and secondary ILs.

#### 2.2.1. Alkylation Reactions

**Figure 3:**
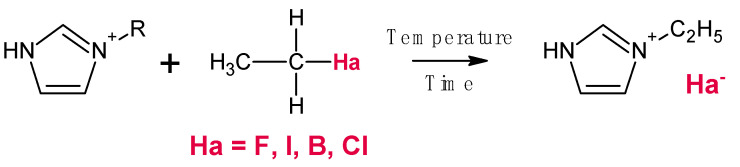
Typical synthesis of the primary ILs by alkylation. Reprinted from Reference [123] under the license CCBYNCND 3.0. Copyright 2015 Longdom.

Generally, this method is used for synthesis of ILs containing halide anions (Cl^−^, Br^−^, I^−^, and F^−^). In this reaction ILs are produced through the alkylation of a base with a haloalkane (Figure 3) [122,123,124]. The low cost of haloalkanes and low reaction temperature are the main benefits of this route [122,123]. The order of haloalkane reactivity rises from chloride to iodide (chloride < bromide < iodide) [122]. For example, 1-chlorobutane needs more time for completion of the reaction than 2-bromobutane at the same operation conditions because bromide is more reactive than chloride [122,123]. Moreover, since bromide and iodide haloalkanes are very reactive, the reaction does not require high temperature (e.g., 1-bromobutane and iodomethane required 35 and 0 °C, respectively) [122]. In the case of iodoalkanes, the temperature should be low in order to prevent the secondary reactions [122].

Furthermore, iodoalkanes are photoactive, and they can easily react with sunlight; as a result, in this case, the reactions are typically conducted in an ice bath and protected from sunlight [122]. The use of haloalkanes comprising fluorine anion is impossible as the chemical bond between fluorine and carbon is very strong; thus, using them for synthesis of ILs are practically impossible [122]. Bao et al. [125] synthesized 1-hexyl-3-methylimidazolium chloride ((HMIm)Cl) via alkylation reaction in order to modify thermal stability and ionic conductivity of PEMFC. 1-methylimidazole, cyclohexane, and 1-chlorohexane were used as base, solvent, and haloalkane, respectively. The reaction was taken place in three-neck round bottom flask at 60 °C for 72 h under stirring. The resultant product was washed with ethyl acetate to remove by-products and unreacted compounds. Eventually, (HMIm)Cl was dried in oven at 70 °C overnight to evaporate the solvent. The hybrid membrane composed of sulfonated poly(2,5-benzimidazole)/montmorillonite/(HMIm)Cl demonstrated the maximum amount of conductivity (4.0 × 10^−2^ S/cm at 220 °C). In another research, Fang et al. [126] synthesized two types of ILs by alkylation reaction: 1-vinyl-3-butylimidazolium bromide ((VBI)Br) and 1-vinyl-3-methylimidazolium iodide ((VMI)I). 1-vinylimidazole, 1-bromobutane, and methyl iodide were the reactants of these two reactions. Since the methyl iodide is much more reactive than 1-bromobutane, methyl iodide required lower operating temperature and stirring time (0 °C and 6 h for (VMI)I, and 35 °C and 12 h for (VBI)Br. After the reaction completion both products were washed with ethyl ether for the by-products removal and then dried at room temperature for 6 h.

Alkylation reaction is used for the primary ILs synthesis, which is useful for production of secondary ILs. As a result, this reaction is an important complementary to acid-base neutralization and metathesis reactions.

#### 2.2.2. Metathesis Reactions

**Figure 4:**
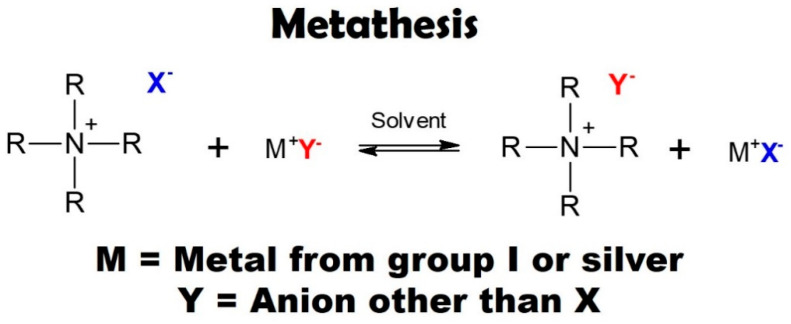
Synthesis of secondary ILs via metathesis reaction. Reprinted with permission from Reference [123]. Copyright 2016 Elsevier.

Metathesis reaction is one of the most common and simplest methods for synthesis of secondary ILs [122,123,124]. In general, the reaction takes place between a metal salt (containing Ag or group I metal in the periodic table) and a primary IL to synthesize halide free ILs (Figure 4) [123,124]. In this reaction, the IL should be separated from impurities (by-products) via phase transfer procedure [122]. There is an anion exchange reaction between metal salt and primary IL which leads to desired IL [89,122]. Hooshyari et al. [127] synthesized a dicationic IL via metathesis reaction, i.e., 1,6-di(3-methylimidazolium) hexane bis(hexafluorophosphate) (DC_6_). The synthesis of this IL consists of two steps. At first, 1,6-di(3-methylimidazolium) hexane bis bromide was obtained by alkylation reaction between 1,6-dibromopropane and 1-methylimidazole. Then, DC_6_ was synthesized by a metathesis reaction between 1,6-di(3-methylimidazolium) hexane bis bromide (IL) and potassium hexafluorophosphate (salt). Lin et al. [128] made 1-vinyl-3-butylimidazolium bis(trifluoromethylsulfonyl)-imide ((VBIm)(NTf_2_)) by using alkylation, and metathesis reactions. Firstly, 1-vinyl-3-butylimidazolium bromide ((VBIm)Br) was prepared by alkylation reaction between *N*-vinylimidazole and 1-bromobutane. Then, (VBIm)(NTf_2_) was synthesized via the metathesis reaction between (VBIm)Br, and bistrifluoromethanesulfonimide lithium (salt).

#### 2.2.3. Acid-Base Neutralization

**Figure 5:**
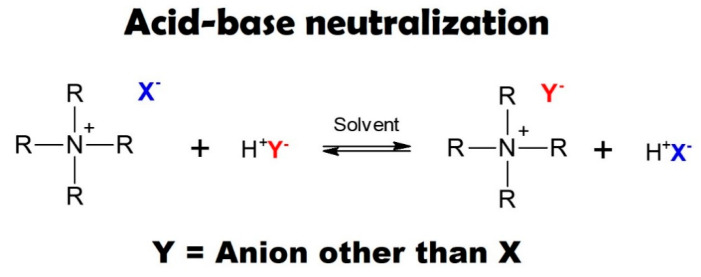
Synthesis of secondary ILs via acid-base neutralization reaction. Reprinted with permission from Reference [123]. Copyright 2016 Elsevier.

Acid-base neutralization is an effective way to synthesize secondary ILs [89]. The basis of this reaction is the neutralization between a Brønsted acid and a Brønsted base, which allows the production of a Pr-IL [78,123]. As highlighted in Figure 5, an anion exchange reaction between the halide anion of the primary ILs and acid anion takes place in the presence of solvent [122,123]. Maiti et al. [129] synthesized 2,3-dimethyl-1-butyl imidazolium dihydrogen phosphate ((MBuIm)(H_2_PO_4_)) by acid-base neutralization method. At first, 2,3-dimethyl-1-butyl imidazolium bromide was formed through the reaction between 1,2-dimethyl imidazole and 1-bromobutane (by alkylation reaction). Finally, (MBuIm)(H_2_PO_4_) was obtained through the acid-base neutralization reaction in the presence of 2,3-dimethyl-1-butyl imidazolium bromide, acetonitrile, and phosphoric acid (H_3_PO_4_). Lin et al. [72] synthesized 1-methylimidazolium trifluoromethanesulfonate ((MIm)(TfO)) via acid-base neutralization. 1-methylimidazole, trifluoromethanesulfonic acid, and ethyl acetate were used as Brønsted base, Brønsted acid, and solvent. For this purpose, 1-methylimidazole was dissolved in ethyl acetate at 0 °C. Then, trifluoromethanesulfonic acid was added drop-wisely to the mentioned solution, and anion exchange reaction took place between base and acid. The resultant IL was dried in a vacuum oven in order to evaporate solvent. However, metathesis and acid-base neutralization reaction have some demerits [123]. As for metathesis reaction, since halide ions are very reactive, they can easily react with water, silver, and other chemical compounds [123]. Another disadvantage is the fact that the number of metal salts available on the market is limited [123]. Concerning acid-base neutralization reaction, hydroxide quaternary cations should be used in the case of weak acids (weaker than hydrohalic acids) [123].

### 2.3. ILs Applications

The widespread use of ILs in science and industry is an inevitable phenomenon owing to tunable features of these organic salts [23,79]. Not only do these chemical compounds improve the efficiency of the processes, but they also do not generate any considerable contamination [23,78]. As a result, ILs are used for various purposes, such as electrochemistry, solvent, engineering, physical chemistry, gas separation, analytical chemistry, gas separation, metal extraction, biological aid, and engineering chemistry [78]. Actually, the exploitation of various ILs can improve the electrochemical properties of fuel cells significantly [126,130,131]. Here, some examples of using ILs in some types of fuel cells are shown. Elumalai et al. [132] prepared hybrid membranes containing 1-methyl-3-(3-trimethoxysilylpropyl)imidazolium chloride (IL)-titanate nanotubes (TnT)/quaternary ammonium functionalized polysulfone (QAPSU) in order to use in AFC. The resultant hybrid membranes demonstrated that, owing to the synergetic effect of IL and TnT, the addition of this hybrid compound increased hydroxyl conductivity, water content, and ion exchange capacity (IEC). Among all hybrid membranes, the sample possessing 5 wt% IL-TnT presented the highest power density (302 mW/cm^2^), water uptake (15.97%), IEC (1.93 (meq/g), and conductivity (2.08·10^−2^ S/cm). In another research, Elumalai et al. [133] fabricated 1-methyl-3-(3-trimethoxysilylpropyl)imidazolium chloride (IL)-Santata Barbara amorphous-15 (SBA-15)/ quaternary polysulfone (QPSU) composite membranes via solvent casting method in order to improve the AFC performance. It was found that the best value of open circuit potential (OCP), power density, water absorption, IEC, and conductivity was for composite sample with 3 wt% IL-SBA-15 (0.87 V, 278 mW/cm^2^, 15.87%, 1.86 meq/g, and 1.89·10^−2^ S/cm, respectively), in which these parameters were much better than membrane without IL (0.65 V, 160 mW/cm^2^, 5.73%, 0.69 meq/g, and 0.71·10^−2^ S/cm, respectively). In addition, ILs are widely used in MFC processes. Hernández-Fernández and co-workers [134] produced supported IL/polyvinylchloride (PVC) hybrid membranes for improvement of MFC efficiency. Two types of ILs were used in that study: methyl trioctil ammonium chloride (MTOA)(Cl), and 1-octyl-3-methylimidazolium hexafluorophosphate (OMIm)(PF_6_). The results displayed that ILs-based membranes could improve proton conduction through the electrolyte. The composite membrane with 50% and 70% *w*/*w* (MTOA)(Cl) showed the acceptable power of 400 and 450 mWm^−3^. Furthermore, the amount of chemical organic demand removal (COD_R_) for the composite membrane comprising 70% *w*/*w* (MTOA)(Cl) was around 80%. Moreover, Hernández-Fernández et al. [135] also found that supported ionic liquid membranes (SILMs) could play a crucial role in transporting of protons through the membrane in MFC processes for treatment of effluents. Various types of SILMs (cations, and anions) were used, and their performances were compared with commercial membranes, including Nafion^®^ and Ultrex^®^. It is worth noting that the membrane possessing methyl trioctyl ammonium chloride (MTOA)(Cl) indicated maximum power and COD_R_ of 103.9 mW/m^3^ and 89.1%, respectively. Thus, it can be seen that the utilization of ILs would result in boosting the performance of MFCs. ILs have been used in order to improve performance and efficiency of PEMFCs. For example, Guerreiro da Trindade et al. [136] fabricated SPEEK based-membranes improved by 1-butyl-3-methylimidazolium tetrafluoroborate ((BMIm)([BF_4_)). Lab-made membranes were immersed into the solution possessing (BMIm)([BF_4_) for various time intervals. It was revealed that the membrane which was dipped for 2 min showed the best thermal stability among others since, at this time, SO_3_^−^ groups can interact with (BMIm)^+^ cations in the best way. This sample also indicated the maximum proton conductivity of 1.0 mS/cm at 100 °C which was 114 greater than for pure SPEEK membrane. During the PEMFC’s exploitation, the highest values for power and current density were 0.13 W/cm^2^ and 0.54 A/cm^2^, respectively. Although (BMIm)([BF_4_) does not hold functional groups, such as hydroxyl, carboxyl, sulfate, and phosphate, in its structure, it resulted in enhancing performance of the SPEEK-based membranes especially with regard to proton conductivity. Furthermore, Yang et al. [137] manufactured PA/Nafion hybrid membranes which were modified by incorporation of 1-butyl-3-methylimidazolium (an ionic liquid cation (ILC)). The incorporation of ILC led to reducing the methanol permeability of Nafion membranes and increasing the PA doping. Moreover, the highest proton conductivity for ILC/PA/ Nafion hybrid membranes was 10.9 mS/cm at 160 °C without humidification. It was found that the usage of PA into the hybrid samples not only improved the thermal stability (up to 250 °C), but it also maintained the mechanical strength at reasonable values (between 2.5 and 9.0 MPa at 160 °C). Therefore, it can be seen that the fabrication of Nafion-based membranes with good potential for using at elevated temperatures is practical by adding ILC. Ye et al. [138] prepared 1-propyl-3-methylimidazolium dihydrogen phosphate ((PMI)(H_2_PO_4_))/H_3_PO_4_/PBI by solution casting method in order to obtain HT-PEMFC. The resultant membrane showed good proton conductivity of 2.0·10^−3^ S/cm at 150 °C and under aqueous-free condition. Moreover, the performance of mentioned composite membrane was investigated under various relative humidity situation (0%, 10%, and 20% RH) at 80 °C. The results demonstrated that, by increasing the humidity, the proton conductivity of (PMI)(H_2_PO_4_)/H_3_PO_4_/PBI membrane was enhanced, and the maximum value for proton conductivity was 1.31 mS/cm at 80 °C under relative humidity of 20%. Although lower humidity is desirable for HT-PEMFCs, owing to the evaporation of water during the separation process, humidity is an important factor for application of PEMFCs at low and moderate temperatures. In another study, Malis et al. [139] fabricated four composite membranes by two polymers (Nafion and poly(vinylidenefluoride-co-hexafluoropropene)) and two ILs (1-butyl-3-methylimidazolium trifluoromethanesulfonate ((BMIm)(TfO)) and 1-ethylimidazolium trifluoromethanesulfonate ((EIm)(TfO))) via solution casting technique. The proton conductivity of membranes were measured under both humid (8%, 17%, and 22% RH) and anhydrous conditions. The proton conductivity of membranes under anhydrous condition were enhanced by increasing the temperature and the maximum amount of this factor was for (BMIm)(TfO)/Nafion membrane (2.25 S/m at 160 °C). The conductivity of (BMIm)(TfO)/Nafion membrane was also measured at 110 °C under 22% RH, and it was around 8.0 S/m. Therefore, it is observed that, by decreasing the temperature and increasing the humidity, the conductivity of composite membrane was significantly increased.

## 3. Application of ILs in PEMFC at Elevated Temperatures

Due to the high ionic conductivity and negligible vapor pressure of ILs, these types of organic salts are able to be employed at middle and elevated temperatures under anhydrous conditions without any sensible issues [140]. Protons (H^+^) in this condition pass through the membrane via Grotthuss mechanism [43]. In this review, FILs containing different kinds of ion exchange groups, including sulfonate, sulfate, phosphate, and imide groups, are discussed because these functional groups can provide a better functionality, affinity, and separation performance for the PEM. Although ILs comprising halides (F^−^, I^−^, Br^−^, and Cl^−^) have some merits, scientists are often looking for new ILs with more functionality and reactivity. A wide range of research studies have been carried out concerning the use of FILs in PEMFC so far, and several of them are presented in this review (Table 2).

### 3.1. ILs Containing Sulfonate and Sulfate Groups

It can be observed that ILs composed of sulfonate and sulfate ion exchange groups have attracted much attention, thanks to their special thermochemical characteristics [139]. There is a vast number of FILs possessing sulfonate, as well as sulfate group, such as 3-triethylammonium hydrogen sulfate, 1-butylimidazole hydrogen sulfate, 1-butyl-3-methylimidazole methanesulfonate, 1-butyl-3-methylimidazolium hydrogen sulfate, 1-methylimidazolium hydrogen sulfate, imidazolium hydrogen sulfate, diethylethylammonium trifluoromethanesulfonate, *N*-ethylimidazolium trifluoromethane-sulfonate, 1-methylimidazolium trifluoromethanesulfonate, 1-butyl-3-methylimidazolium trifluoromethanesulfonate, and 1-ethylimidazolium trifluoromethanesulfonate [139,141,142,143,144,145]. Chen et al. [141] elaborated SPEEK-based PEMs which were modified by ILs (1-ethyl-3-methylimidazole tetrafluoroborate (EB) or 1-butyl-3-methylimidazole methanesulfonate (BS)) and yttrium oxide (Y_2_O_3_). The resultant membranes were prepared via casting solution method, and the influence of the ILs and Y_2_O_3_ content on the membranes properties was investigated. The results illustrated that the conductivity of membranes was increased by raising the temperature from 30 to 90 °C, although pristine SPEEK membrane showed a decreasing trend by increasing temperature. The sample containing SPEEK/BS/Y_2_O_3_ displayed much higher conductivity than SPEEK/EB/Y_2_O_3_ at 90 °C under both 100% and 50% relative humidity (RH) (1.18·10^−1^ S/cm and 1.02·10^−1^ S/cm for SPEEK/BS/Y_2_O_3_ and 9.04·10^−2^ S/cm and 8.04·10^−2^ S/cm for SPEEK/EB/Y_2_O_3_, respectively). Besides, it was found that incorporation of ILs and Y_2_O_3_ improved the thermal stability and water uptake of membranes. The tensile strength for SPEEK/EB/Y_2_O_3_ and SPEEK/BS/Y_2_O_3_ was 2.61 MPa and 2.33 MPa, respectively. Owing to the BS, the proton conductivity of membrane comprising BS is slightly higher due to the presence of sulfonate group (hydrophilic functional group) in its structure. Moreover, all membranes revealed excellent thermal stability between 250 and 350 °C due to the interaction between SO_3_^−^ anions of SPEEK and ILs. The resultant composite membranes demonstrated promising potential for using at middle and elevated temperatures. Guerreiro da Trindade et al. [142] produced SPEEK/polybenzimidazole (PBI)/ILs membranes by casting technique (pouring the polymeric solution in a Petri dish, then drying at oven under vacuum). The influence of two kinds of ILs (3-triethylammonium hydrogen sulfate ((TEA-PS)(HSO_4_)) and 1-butylimidazole hydrogen sulfate ((BImH)(HSO_4_))) on the membrane properties was studied at high temperatures and low RH. It was noticeable that the oxidative stability was improved by raising the PBI content, while the membrane proton conductivity was improved by the increasing of the ILs quantity. Additionally, the composite membrane with 10 wt% PBI and 5 wt% (TEA-PS)(HSO_4_) and 2.5 wt% (BIm)(HSO_4_) showed the highest conductivity value compared to the other membranes. It was also found that the composite membranes with 2.5 and 5 wt% (TEA-PS)(HSO_4_) were demonstrated the highest thermal stability (between 300 and 400 °C). The composite membrane comprising SPEEK/PBI/(TEA-PS)(HSO_4_) with 5 wt% (TEA-PS)(HSO_4_) showed the highest OCP value (0.97 V), current density (1.83 A/cm^2^), and power density (0.41 W/cm^2^) at 100 °C without any reactant diffusion limitations, in comparison with other membranes which suffered from electrode poisoning, short-circuit, and gas crossover. However, the composite membrane containing (BImH)(HSO_4_) displayed lower OCP, current density and power density compared to even pure SPEEK membrane. Guerreiro da Trindade et al. [143] obtained SPEEK/ILs (1-butyl-3-methylimidazolium hydrogen sulfate ((BImH)(HSO_4_)), 1-methylimidazolium hydrogen sulfate ((MI)(HSO_4_)), and imidazolium hydrogen sulfate (Im)(HSO_4_)) membranes by casting method. The results revealed that the highest proton conductivity was obtained for SPEEK/(MI)(HSO_4_) with 5 wt% of IL at two different conditions (at 25 °C and 100% RH and 80 °C and 80% RH) equal to 120 and 150 mS/cm, respectively. These values are much higher than the value for pristine SPEEK membrane (78 and 101 mS/cm at 25 °C and 100% RH and 80 °C and 80% RH, respectively). Furthermore, SPEEK/(BMI)(HSO_4_) membrane with 5 wt% 1-butyl-3-methylimidazolium hydrogen sulfate exhibited the most promising results concerning current density value and power density at 100 °C (2.33 A/cm^2^ and 0.53 W/cm^1^, respectively). The highest amount of water uptake (87.6%) was reported for SPEEK/(Im)(HSO_4_) membrane sample (1 wt% IL). Generally, the addition of these three ILs led to improving the oxidative stability and the best improvement was found for the membrane with (MI)(HSO_4_). Additionally, hybrid membranes were thermally stable up to 200 °C. Li et al. [144] elaborated inorganic-organic composite membranes based on SPEEK/silica/diethylethylammonium trifluoromethanesulfonate ((dema)(OTf)) via sol-gel technique for HT-PEMFC. It was observed that these hybrid composite membranes indicated higher thermal stability (up to 250 °C) as compared with the unmodified membrane. It was also found that using silica in the composite samples could improve the membranes flexibility and mechanical strength. The maximum proton conductivity was reported for the membrane containing 50 wt% (dema)(OTf) (2.0 × 10^−2^ S/cm at 220 °C under the dry environment). Lin et al. [145] produced protic ionic liquid (Pr-IL)/silica nanomaterial/poly(styrene-co-acrylonitrile) (SAN) hybrid membranes by casting solution-photo cross-linking method in order to utilize at elevated temperature and anhydrous condition. The name of IL was *N*-ethylimidazolium trifluoromethane-sulfonate ((EIm)(TfO)). It was noticeable that hybrid samples demonstrated high thermal stability up to 300 °C. All hybrid membranes showed reasonable flexibility, transparency, and mechanical stability (the values for strong module were between 5 and 11 MPa at 120 °C). The use of optimum content of silica inorganic compound allowed rising the proton conductivity as this filler provided many networks and channels for proton conduction. The resultant (EIm)(TfO)/silica/SAN hybrid membranes showed the maximum proton conductivity of 1 × 10^−2^ S/cm at 160 °C under dry conditions. Lin et al. [72] prepared Pr-IL-based composite membranes via casting solution-photo cross-linking for applying at high temperatures. In that case, 1-(3-aminopropyl)-3-methylimidazolium bromide modified with graphene oxide ((APMIm)(Br)—GO), and 1-methylimidazolium trifluoromethanesulfonate ((MIm)(TfO)) were used as dopant and proton carrier in PEM at elevated temperatures, respectively. The resultant hybrid samples illustrated great mechanical (tensile strength) and thermal stability (34.2 MPa and 300 °C, respectively). It was found that the application of (APMIm)(Br)—GO into the membranes led to increasing proton conductivity, and the hybrid membrane possessing 1.0 wt% (APMIm)(Br)—GO displayed the maximum proton conductivity of 1.48 × 10^−2^ S/cm at 160 °C. Furthermore, hybrid samples containing (APMIm)(Br)—GO indicated much better retention ability of IL than pristine one. It was also observed that, by enhancing the amount of (APMIm)(Br)—GO from 0.3 to 1.2 wt%, the tensile strength value increased from 17 to 34 MPa. Malis et al. [139] elaborated composite membranes based on different polymers (i.e., poly(vinylidene fluoride (PVDF)-co-hexafluoropropene (HFP)) and Nafion) and ILs (1-butyl-3-methylimidazolium trifluoromethanesulfonate (BMIm)(TfO) or 1-ethylimidazolium trifluoromethanesulfonate (EIM)(TfO)) by solution casting method to use in high temperature fuel cells. The resultant composite samples showed great performance under both hydrous and anhydrous conditions. The maximum amount of conductivity under humid condition was for 8.5 S/m at 110 °C and 22% RH for (EIM)(TfO)/PVDF-co-HFP composite membrane. On the one hand, under dry conditions, the membrane proton conductivity was enhanced by raising the temperature, and the highest value of 2.25 S/m at 160 °C was obtained for (BMIm)(TfO)/Nafion composite membrane. Under humid conditions, the proton conductivity of samples was reduced at temperatures higher than 100 °C because of the water evaporation from membranes. Furthermore, the highest value of power density (1.2 mW/cm^2^) was achieved for (BMIm)(TfO)/Nafion composite membrane. Hence, these results revealed that (BMIm)(TfO)/Nafion composite membrane is rather promising for its use at high temperatures without humidification.

### 3.2. ILs Possessing Imide Group

FILs with imide group is widely use in PEMs due to their high separation efficiency [128]. Among such ILs, one can note 1-H-3-methylimidazolium bis(trifluoromethanesulfonyl)imide, 1-methylimidazolium bis(trifluoromethylsulfonyl)imide, 1-ethylimidazolium bis(trifluoromethylsulfonyl)imide, 1-propylimidazolium bis(trifluoromethylsulfonyl)imide, 1-butylimidazolium bis(trifluoromethylsulfonyl)imide, and 1-vinyl-3-butylimidazolium bis(trifluoromethylsulfonyl)-imide, and 1,3-di(3-methylimidazolium) propane bis(trifluoromethylsulfonyl)imide [127,128,146,147,148]. For example, Lin et al. [128] fabricated phosphoric acid-doped composite membranes using hydrophobic 1-vinyl-3-butylimidazolium bis(trifluoromethylsulfonyl)-imide ((VBIm)(NTf_2_)) via casting solution method, followed by photo cross linking (by means of UV irradiation). It was found that the phosphoric acid uptake was initially increased (up to 137.74%) with the (VBIm)(NTf_2_) content increasing and then reduced. The resultant samples exhibited high proton conductivity (10^−2^ S/cm at 180 °C) and thermal stability (between 300 and 350 °C) under anhydrous environment. Lin et al. also claimed that mechanical stability of membranes was improved (up to 18.01 MPa) with the increasing of IL quantity owing to its hydrophobic nature. Additionally, the highest proton conductivity (4.14×10^−2^ S/cm at 180 °C) was reported for (VBIm)(NTf_2_)_35_-VIm_35_ (35 wt% (VBIm)(NTf_2_) and 35 wt% VIm). Hooshyari et al. [148] prepared two PBI-based membranes doped by monocationic ionic liquid: 1-hexyl-3-methylimidazolium bis(trifluoromethanesulfonyl)imide (PMC_6_), and, by dicationic ionic liquid: 1,3-di(3-methylimidazolium)propane bis(trifluoromethylsulfonyl)imide (PDC_3_), in order to use at high temperature without the water presence. The membranes containing PA/dicationic ILs showed higher value of thermal stability, proton conductivity, and fuel cell performance than PA/monocationic composite samples. In fact, the number of charge groups in PDC_3_ is much important as compared with PMC_6_, thus leading to more developed functional network for the proton conduction through the membrane at high temperatures and under anhydrous conditions. It was found that the proton conductivity of composite membranes containing PA/PBI, PA/PMC_6_/PBI, and PA/PDC_3_/PBI demonstrated an increasing trend by increasing the temperature up to 180 °C. The highest proton conductivity of 81 mS/cm at 180 °C was obtained for PA/PDC_3_/PBI composite membrane under non-humid environment. Moreover, the composite sample composed of dicationic IL showed a great power density of 0.44 W/cm^2^. In addition, the composite membranes with PDC_3_ revealed higher mechanical stability than composite membranes containing PMC_6_. Fatyeyeva et al. [146] elaborated polyimide (PI)/protic ionic liquid (Pr-IL) hybrid membranes for employing in middle and elevated temperature PEMFCs. In that study, four Pr-ILs with different cations were considered. It was noticeable that the synthesized Pr-ILs showed an excellent thermal stability between 360 and 400 °C. Moreover, it was found that the most important criteria with regard to proton conductivity was the increase of temperature rather than the type of IL cation. Furthermore, PI/Pr-IL composite membranes exhibited the highest proton conductivity of 10^−3^ S/cm at 160 °C. These elaborated membranes demonstrated that incorporation of functionalized Pr-ILs possessing imide group facilitated PEMFCs application at middle and elevated temperatures. Van de Ven et al. [147] prepared PBI-based membranes which were impregnated by 1-H-3-methylimidazolium bis(trifluoromethanesulfonyl)imide ((h-mim)(Ntf_2_)). The resultant composite membranes displayed the highest value for proton conductivity of 1.86 mS/cm. Furthermore, the maximum power density of 0.039 W/cm^2^ was obtained at 150 °C. Moreover, PBI/(h-mim)(Ntf_2_) composite membranes demonstrated good thermal stability at 190 °C. So, this composite membranes could be regarded as good candidates for HT-PEMFC due to the presence of both (h-mim)(Ntf_2_) containing imide group, but also PBI with excellent thermal stability.

### 3.3. ILs Comprising Phosphate Group

A wide range of FILs possessing phosphate and phosphonate groups were studied and used on membranes with improved physical and chemical properties, such as tertiary amine phosphate, 1-n-butylimidazolium bis(2-ethylhexyl)phosphate, 1-n-butylimidazolium dibutylphosphate, 1-n-methylimidazolium dibutylphosphate, 1-butyl-3-ethylbenzimidazolium dihydrogen phosphate, and 2-hydromethyl) trimethylammoniun dimethyl phosphate [149,150,151,152,153,154]. Ke et al. [149] prepared polypropylene (PP)-nonwoven (NW)/tertiary amine phosphate (PP-NW/(N_111_)(H_2_PO_4_)) composite membrane via a reciprocating rolling process for HT-PEMFC. Such lab-made membranes indicated good current density (600 mA/cm^2^ at 0.1 V and 140 °C, and under anhydrous environment). Furthermore, performed OCP decay acceleration test of PP-NW/(N_111_)(H_2_PO_4_) membranes revealed an acceptable stability. In addition, the measured ionic conductivity of PP-NW/(N_111_)(H_2_PO_4_) membrane was 0.016 S/cm at 160 °C. Elumalai et al. [150] fabricated SPEEK/phosphonate ionic liquid (PIL)-SBA-15 composite membranes via solution casting method. The water uptake value of the composite samples was enhanced by increasing the concentration of PIL-SBA-15. In addition, proton conductivity and IEC of the composite membrane with 6 wt% PIL-SBA-15 were higher than other membranes (10.2 mS/cm^1^ at 140 °C and 2.56 meq/g, respectively). It was also found that the highest value of power density was 183 mW/cm^2^ at 140 °C. The composite membrane comprising 6 wt% PIL-SBA-15 exhibited the most promising results regarding proton conductivity (10.2 mS/cm at 140 °C) and mechanical stability (23 MPa). The improved membrane behavior was explained by the hydrophilic nature of phosphonate anion group (H_2_PO_4_^−^). Kowsari et al. [151] produced 1-methylimidaolium dihydrogen phosphate ((MIm)(H_2_PO_4_))-co-GO/SPI composite membranes as polymer electrolyte membrane to improve the PEMFC. It was found that the maximum proton conductivity under both 40% and 80% RH were reported for composite membrane with 5 wt% of (MIm)(H_2_PO_4_)-co-GO (77.2 mS/cm at 160 °C, and 124.3 mS/cm at 120 °C). It was also found that all composite membranes showed a higher value for water uptake than the pure SPI sample and the membrane with 5 wt% of IL-co-GO displayed maximum water uptake of 47.3% owing to the hydrophilic nature of IL. In addition, it was shown that all samples showed a good thermal stability up to 275 °C. Maiti et al. [129] fabricated GO/dihydrogen phosphate functionalized ionic liquid (FIL-H_2_PO_4_)/Nafion composite membrane via casting solution technique. 2,3-dimethyl-1-butyl imidazolium dihydrogen phosphate ((DMBuIm)(H_2_PO_4_)) was used as IL. The resultant composite membranes demonstrated that the incorporation of GO/(DMBuIm)(H_2_PO_4_) as modifiers into the polymer membranes allows increasing the thermal stability. It was found that the proton conductivity was enhanced considerably by temperature and (DMBuIm)(H_2_PO_4_) content increasing. Proton conductivity measurements revealed that the highest value of 0.061 S/cm at 110 °C was obtained for the composite membrane. This value is 1.3 time higher than the value of commercial Nafion membrane. Besides, the best performance was indicated for GO/(DMBuIm)(H_2_PO_4_)/Nafion composite membrane with a power density of 0.02 W/cm^2^ at 110 °C. Dahi et al. [152] produced PI/IL composite membranes via phase inversion technique. Supported ionic liquid membranes (SILM) were prepared via the Matrimid^®^ membrane impregnation with three Pr-ILs, including 1-n-butylimidazolium bis(2-ethylhexyl)phosphate ((C_4_im)(BEHP)), 1-n-butylimidazolium dibutylphosphate ((C_4_im)(DBP)), and 1-n-methylimidazolium dibutylphosphate ((C_1_im)(DBP)). It was noticeable that the proton conductivity of obtained membranes was increased by increasing the temperature on the contrary to Nafion membranes. Moreover, the SILM containing (C_4_im)(DBP) exhibited the best performance of proton conductivity—2.0·10^−2^ S/cm at 115 °C. Hernández Carrillo et al. [153] fabricated poly(2,5-benzimidazole) (ABPBI)/PA/1-butyl-3-ethylbenzimidazolium dihydrogen phosphate (BEBzIm)(H_2_PO_4_) composite membranes. The resultant samples presented the enhanced thermal stability with the (BEBzIm)(H_2_PO_4_) addition. It was also found that the best value for conductivity was 9.02·10^−4^ S/cm at 150 °C. The performance of these composite membranes indicated that the use of PA/(BEBzIm)(H_2_PO_4_) possessing a number of functional groups could improve proton conductivity and thermal stability. Eguizábal et al. [154] manufactured conductive composite membranes with Pr-IL/zeolite/PBI/PA by solution casting method. In fact, three types of Pr-ILs, including 2-hydromethyl trimethylammoniun dimethyl phosphate (Pr-IL_1_), *N*,*N*-dimethyl-*N*-(2-hydroxyethyl) ammonium bis(trifluoromethanesulfonyl)imide (Pr-IL_2_), and 1-H-3-methylimidazolium bis(trifluoromethanesulfonyl)imide (Pr-IL_3_), were encapsulated into the zeolite pores (NH_4_BEA and NaY). It was found that, among all composite samples, the membrane with Pr-IL_3_-NaY exhibited the most promising results for HT-PEMFC application—54 mS/cm at 200 °C for the membrane with 3 wt% Pr-IL_3_/NaY/PBI. Besides, this membrane demonstrated much better H^+^/H_2_ transport selectivity than pure PBI, and PBI/NaY membranes. Additionally, the composite membrane with 3 wt% Pr-IL_3_/NaY/PBI showed good thermal and chemical stability necessary for the long-term membrane utilization. Therefore, Pr-IL_3_/NaY/PBI/PA composite sample clearly demonstrated that it can be the best choice for the PEMFC operation at elevated temperature.

A summary regarding the utilization of FILs into the PEMs at middle and elevated temperatures under various humid conditions is provided in Table 2.

**Table 2:** The influence of different types of FILs on PEM performance.

Type of FIL	Membrane Compositions	Preparation Technique	Results	Ref.
Sulfonate and sulfate	EB/Y_2_O_3_/SPEEK BS/Y_2_O_3_/SPEEK	Solution casting	Both ILs improved thermal stability (up to 250–350 °C), and water uptake of composite membranes. BS/Y_2_O_3_/SPEEK composite membrane showed the highest conductivity at 90 °C and at 50% and 100% RH. EB/Y_2_O_3_/SPEEK composite sample demonstrated the highest mechanical stability of 2.61 MPa.	[141]
	(TEA-PS)(HSO_4_)/PBI/SPEEK (BImH)(HSO_4_)/PBI/SPEEK	Solution casting	Oxidative stability, and proton conductivity were increased by addition of PBI/IL into the membrane. The composite membranes demonstrated good thermal stability between 300 and 400 °C. (TEA-PS)(HSO_4_)/PBI/SPEEK membranes with 2.5, and 5 wt% IL showed the highest thermal stability and the highest OCP, current density, and power density of 0.97 V, 1.83 A/cm^2^, and 0.41 W/cm^2^, respectively.	[142]
	(BMI)(HSO_4_)/SPEEK (MI)(HSO_4_)/SPEEK (Im)(HSO_4_)/SPEEK	Solution casting	The thermal stability was improved up to 200 °C. The usage of ILs led to enhancing oxidative stability (MI)(HSO_4_)/SPEEK membrane displayed the highest proton conductivity of 150 mS/cm. (BMI)(HSO_4_)/SPEEK sample showed the highest current, and power density of 2.33 A/cm^2^ and 0.53 W/cm, respectively.	[143]
	(dema)(OTf)/silica/SPEEK	Sol-gel	The composite membranes were studied at elevated temperature and under anhydrous environment. The composite membranes showed good thermal stability (250 °C). The proton conductivity was improved up to 2.0·10^−2^ S/cm at 220 °C and under the dry condition. The results presented that the addition of silica could improve the flexibility and mechanical properties.	[144]
	(EIm)(TfO)/silica/poly(styrene-co-acrylonitrile)	Solution casting followed by photo cross-linking	The resultant membranes were thermally stable up to 300 °C. The hybrid samples presented good mechanical stability. The proton conductivity of hybrid membrane was 1·10^−2^ S/cm at 160 °C under anhydrous condition.	[145]
	(MIm)(TfO)/(APMIm)(Br)-GO/poly(styrene-co-acrylonitrile)	Solution casting followed by photo cross-linking	The usage of IL increased significantly thermal stability up to 400 °C. The maximum proton conductivity was noted at 1.48·10^−2^ S/cm at 160 °C. The hybrid samples showed better retention ability of IL than pure membrane.	[72]
	(BMIm)(TfO)/Nafion (BMIm)(TfO)/PVDF-co-HFP (EIm)(TfO)/Nafion (EIm)(TfO])/PVDF-co-HFP	Solution casting	The composite membranes revealed good performance under both hydrous and anhydrous conditions. The highest proton conductivity was obtained at 2.25 S/m at 160 °C with power density of 1.2 mW/cm^2^ for the (BMIm)(TfO)/Nafion composite membrane. (EIm])(TfO)/PVDF-co-HFP composite membrane displayed the highest proton conductivity of 8.5 S/m at 110 °C under 22% RH.	[139]
Imide	(VBIm)(NTf_2_)/H_2_PO_4_^−^/poly(styrene-co-acrylonitrile)	Solution casting followed by photo cross linking	The composite sample showed proton conductivity of 4.14·10^2^ S/cm at 180 °C without humidification. The composite membrane showed good mechanical stability and was thermally stable up to 300 °C.	[128]
	PDC_3_/PA/PBI PMC_6_/PA/PBI	Solution casting	The composite membranes composed of PDC_3_ illustrated higher proton conductivity and thermal stability than the membrane containing PMC_6_. PDC_3_/PA/PBI membrane showed the highest proton conductivity of 81 mS/cm at 180 °C and anhydrous environment. The composite membrane containing PDC_3_/PA/PBI showed excellent power and current density (0.44 W/cm^2^, 0.89 A/cm^2^ at 180 °C and under anhydrous conditions, respectively).	[148]
	(MIm)(TFSI)/Matrimid^®^ (EIm)(TFSI)/Matrimid^®^ (PIm)(TFSI)/Matrimid^®^ (BIm)(TFSI)/ Matrimid^®^	Phase inversion	All composite membranes were thermally stable between 360 and 400 °C. The maximum proton conductivity of 10^−3^ S/cm was obtained at 160 °C.	[146]
	(h-mim)(Ntf_2_)/PBI	Solution casting	The maximum ionic conductivity was 1.86 mS/cm at 190 °C. The highest power density was 0.039 W/cm^2^ was achieved at 150 °C. The lab-made composite membrane indicated great thermal stability up to 190 °C.	[147]
Phosphate	(N_111_)(H_2_PO_4_)/PP-NW	Reciprocating rolling process	The composite home-made membrane presented high current density of 600 mA/cm^2^ at 0.1 V, 140 °C, and under aqueous-free situation. The conductivity of composite sample was improved up to 0.016 S/cm^1^ at 160 °C.	[149]
	Phosphonated IL-SBA-15/SPEEK	Solution casting	Addition of composite content (PIL-SBA-15) resulted in enhancing the water uptake. The maximum value for power density was 183 mW/cm^2^ at 140 °C. The membrane mechanical stability of 23 MPa was obtained. The composite sample with 6 wt% PIL-SBA-15 demonstrated the highest proton conductivity of 10.2 mS/cm^−1^ at 140 °C.	[150]
	(MIm)(H_2_PO_4_)-co-GO/ SPI	Solution casting	The maximum proton conductivity was 0.0772 S/cm at 160 °C. The highest value for water uptake was 47.3% for the sample possessing 5 wt% (MIm)(H_2_PO_4_)-co-GO. The composite membrane was thermally stable up to 275 °C.	[151]
	(DMBuIm)(H_2_PO_4_)/ GO/Nafion	Solution casting	The thermal stability of composite membrane was boosted up to 300 °C. The conductivity of membranes was risen by increasing the temperature and content of (DMBuIm)(H_2_PO_4_). The maximum conductivity was 0.061 S/cm^1^ at 110 °C under non-humidification. The best value for power density was 0.02 W cm^−2^ at 110 °C.	[129]
	(C_4_im)(BEHP)/Matrimid^®^ (C_4_im)(DBP)/Matrimid^®^ (C_1_im)(DBP)/Matrimid^®^	Phase inversion	Unlike Nafion membrane, the proton conductivity of composite samples had a direct correlation with temperature. The composite membrane comprising (C_4_im)(DBP) demonstrated the best conductivity of 2.0·10^−2^ S/cm at 115 °C.	[152]
	(BEBzIm)(H_2_PO_4_)/ABPBI/PA	Solution casting	The addition of (BEBzIm)(H_2_PO_)_ had a strong effect on thermal stability. The highest conductivity was 9.02·10^−4^ S/cm at 150 °C.	[153]

## 4. Leaching of ILs from the Membranes

Generally, notwithstanding the fact that the use of various types of ILs can have a lot of benefits for PEM, including enhancement of thermal stability, improving fuel cell performance, and proton conductivity at elevated temperature and low humidification, the loss of ILs is the main negative point of this approach [155,156,157]. In fact, after some minutes of PEM exploitation, the leakage of IL occurs and causes serious limitations in the IL application in fuel cells [155,158]. As a result, in many studies regarding ILs-based membranes for middle and high temperature PEMFCs, the leaching of ILs has been investigated [156,158]. This weight loss of IL can be calculated by using the following formula [144,156,157,158]:

(1) $$\%Loss=\frac{Wo-Wi}{Wo}\times 100,$$

where in this formula *W_O_* is membrane primary weight, and *Wi* is the weight of membrane after immersion.

A wide range of research studies have been taken up so far in order to investigate ILs leaching and how to deal with this challenging issue [155,156,157,158]. First, the selection of proper ILs in terms of their hydrophilicity and hydrophobicity, and providing the optimum amount of them have positive impacts on the reduction of ILs leakage. Jothi and co-workers [156] produced SPEEK/ethyl-3-methylimidazolium diethyl phosphate ((EMIm)(DEP)) composite membranes via solution casting method for HT-PEMC in aqueous-free environment. It is worth noting that researchers investigated IL leaching phenomenon from the samples containing various concentrations of doped IL in the composite membranes in different regular ranges of time. The resultant SPEEK/(EMIm)(DEP) composite membranes illustrated that the sample possessing 10 wt% IL had the minimum leakage around 9% after 50 min of operation. Other samples comprising 30% and 50% IL showed worse performance with 19% and 28% weight loss, respectively. Besides, the leaching from membranes containing 10% and 30% IL reduced with time and remained stable after 28 h. Actually, they proposed that the primary absorption of IL with the polymer body have strong effect on leaching of IL. Using an extra content of ILs resulted in insufficiently strong bonds between ILs and the polymer background, and, in turn, it can be observed that the composite membranes possessing higher proportion of ILs had greater leaching. Malik and co-workers [157] prepared novel composite membranes composed of sulfonated poly(ether ketone) (SPEK)/aprotic ILs (A-ILs) to utilize for PEMFCs at middle and high temperatures by solution casting. Two imidazolium-based ILs were used, i.e., 1-butyl-3-methyl-imidazolium trifluromethanesulfonate ((bmim)(OTf)), and 1-butyl-3-methyl-imidazolium bis(trifluoromethanesulfonyl)imide ((bmim)(NTf_2_)). The IL leaching was studied as a function of operation temperature, IL nature (hydrophilic or hydrophobic) and content of ILs. The ILs weight loss in all composite membranes at 80 °C were higher than at 25 °C, and the membrane with 70 wt% (bmim)(OTf) demonstrated the highest leakage at 80 °C (around 55%). Moreover, owing to the hydrophilic nature of (bmim)(OTf), the higher leaching was observed compared to the membrane with (bmim)(NTf_2_), which is more hydrophobic. Additionally, the size of (NTf_2_) anion is larger than that one of (OTf) anion, thus reducing the ion movement and, so, leaching from the membranes. Moreover, in all composite membranes, increasing concentration of ILs enhanced the IL leakage. The results showed that in order to obtain a composite membrane with the minimum leaching, an optimal content of hydrophobic ILs is recommended. Additionally, the usage of inorganic compounds (such as silica and alumina) is effective tool to diminish the IL leakage from the membrane. Fernicola and co-workers [158] fabricated ILs/poly(vinyldenefluoride-co-hexafluoropropylene) (PVDF-co-HFP) hybrid membranes with two types of ceramic fillers, i.e., Al_2_O_3_ and SiO_2_d. In this research, *N*-ethylimidazolium bis(trifluoro methane sulfonyl)imide ((EIm)(TFSI)), *N*-methylimidazolium bis(trifluoro methane sulfonyl)imide ((MIm)(TFSI)), and 1-methylpyrrolidinium bis(trifluoro methane sulfonyl)imide ((MPy)(TFSI)) were used. They conducted Fenton and water stability tests in order to determine the membrane weight losses. The results showed that all hybrid samples suffered from the IL leakage. However, the hybrid membrane containing 1-methylpyrrolidinium bis(trifluoromethanesulfonyl)imide ((MPy)(TFSI)) without ceramic incorporation presented the smallest weight loss (around 45%) since 1-methylpyrrolidinium is considered as a strong base which can increase IL stability in membrane and decrease the hydrogen binding impacts. Furthermore, (MPy)(TFSI)/PVDF-co-HFP possessing alumina displayed slightly better IL retention than the membrane with silica (52% and 55%, respectively) due to the fact that Al_2_O_3_ has higher absorbing ability than SiO_2_ owing to dipolar, and hydrogen interactions. Moreover, the sulfonation degree can be also considered as effective approach for the leakage reducing. For example, Li and co-workers [144] elaborate SPEEK/SiO_2_/diethylethylammonium trifluoromethanesulfonate ((dema)(OTf)) inorganic-organic hybrid membranes for HT-PEMFC via sol-gel process. They showed that the IL leaching diminished with the increase of both the degree of sulfonation, and amount of inorganic additive (SiO_2_). In general, increasing of the sulfonation degree caused increased number of hydrophilic and reactive SO_3_^−^ groups; thus, the improved interactions between ILs and SO_3_^−^ can be noted. Additionally, the increase of SiO_2_ quantity provided a number of reactive sites possessing silicon and oxygen which have a sufficient potential to interact with ILs, and such electrostatic interactions can reduce the leaching of ILs. Thus, the results revealed that enhancing sulfonation degree, and the usage of inorganic compounds can play a crucial role in the reduction of ILs leaching. Moreover, the utilization of NPs can have also significant effects on the ILs retention during the process. Lin and coworkers [159] fabricated composite membranes comprising *N*-ethylimidazolium trifluoromethanesulfonate ((EIm)(TfO))/polymerizable oil (acrylonitrile, styrene, and divinylbenzene) 1-methyl-3-((triethoxysilyl)propyl) imidazolium chloride ((TMI)(Cl)) functionalized by silica NPs (Im-Silica) via in situ cross-linking method so as to apply in anhydrous PEMFC. The resultant composite samples revealed that they have not only good proton conductivity under dry environment, but they also have excellent IL retention. The pristine membrane without Im-Silica NPs showed the IL leakage around 90% after few minutes, whereas this value was 70% for composite membrane containing 15 wt% Im-silica NPs. It was also found that after 2 h of the membrane immersion into distilled water, the composite sample with 15 wt% Im-silica NPs revealed the leaching of ILs around 85%, which was better than the pristine membrane (approximately 95%). Besides, the conductivity measurements confirmed the above results, since the composite membrane possessing 15 wt% Im-silica NPs had the maximum conductivity value during first 2 h. In addition, the application of porous inorganic compounds can be also a promising way for decreasing the IL release. Wang and co-workers [160] prepared SPEEK/mesoporous silica/ILs for PEMFC at elevated and under anhydrous condition by casting solution. Different ILs, such as 1-ethylimidazolium trifluoromethanesulfonate ((EIm)(TfO)), diethylmethylammonium trifluoromethane-sulfonate ((dema)(TfO)), 1-butyl-3-methylimidazolium chloride ((BMIm)(Cl)), 1-butyl-3-methylimidazolium trifluoromethanesulfonate ((BMIm)(TfO)), and 1-butyl-3-methylimidazolium tetrafluoroborate ((BMIm)(BF_4_)), were used in the composite membranes. It was found that SPEEK/mesoporous silica/(BMIm)(BF_4_) composite membrane, including 7.5 wt% mesoporous silica and 50 wt% 1-butyl-3-methylimidazolium tetrafluoroborate, demonstrated the maximum proton conductivity of 15 mS/cm at 200 °C without humidification. In addition, all of the composite samples showed acceptable thermal stability between 270 and 400 °C. In order to study the leaching of ILs from the composite membranes, the influences of IL type was tested for the SPEEK/ILs membranes. The obtained results indicated that SPEEK/(BMIm)(BF_4_) had the lowest amount of weight loss (close to 30%). Moreover, for the ILs with the same anion ((TfO)), the order of leakage was as follows: (dema)(TfO) > (EIm)(TfO) > (BMIm)(TfO), thus indicating the cation structure influence. The influence of the silica concentration (7.5% or 10%) was also investigated. It was discovered that the IL leakage is considerably reduced as compared with SPEEK/ ILs composite membranes, whatever the silica concentration was. In fact, the main reason of these results is the formation of bonds between silica (-OH), and ILs. The minimum mass loss was noted for SPEEK/silica/(BMIm)(Cl) membrane with 10 wt% silica and 30 wt% (BMIm)(Cl) due to the fact that this IL can easily enter to the silica pores. In another research study, Chu et al. [155] prepared polyamidoamine (PAMAM) dendrimer-based macromolecular Pr-IL/poly(styrene-co-acrylonitrile) composite membranes by in situ photo-crosslinking technique. (PAMAM G4.0-NH_3_^+^H_2_PO_4_^−^), (PAMAM G4.0-NH_3_^+^HSO_4_^−^), and (PAMAM G4.0-NH_3_^+^Tf_2_N^−^) containing functionalized anions, such as H_2_PO_4_^−^, HSO_4_^−^, and Tf_2_N^−^, were synthesized for this purpose. It was found that the obtained membranes demonstrated an acceptable transparency and flexibility with good thermal stability up to 350 °C. Besides, the maximum proton conductivity was reported for (PAMAM G4.0-NH_3_^+^HSO_4_^−^)-based membrane (1.2·10^−2^ S/cm at 160 °C and under dry situation), which was much greater than for samples with small-molecule Pr-ILs. Thus, the order of conductivity was (PAMAM G4.0-NH_3_^+^HSO_4_^−^) > (PAMAM G4.0-NH_3_^+^H_2_PO_4_^−^) > (PAMAM G4.0-NH_3_^+^Tf_2_N^−^). Moreover, the increased fraction of macromolecular Pr-ILs weigh fraction also increased the membrane conductivity and decreased the IL leaching. The membranes containing hydrophobic ILs demonstrated a better IL retention ability than hydrophilic ones. For example, the membrane composed of (PAMAM G4.0-NH_3_^+^H_2_PO_4_^−^) lost about 90% of IL after 60 min, while this value is only 35% for membrane possessing (PAMAM G4.0-NH_3_^+^Tf_2_N^−^). These results revealed that ion exchange group presence and acceptable hydrophobicity level can be key factors in order to obtain a PEM with appropriate retention ability able to work at elevated temperature. Moreover, coating the membrane with silicon can reduce ILs leaching. Izak et al. [161] prepared multiphase membrane containing ceramic nanofiltration module/tetrapropylammonium tetracyanoborate ((C_3_H_7_)_4_N])(B(CN)_4_))/silicon in order to use in pervaporation process. The membrane was modified by ((C_3_H_7_)_4_N)(B(CN)_4_) and then coated by silicon. The resultant membrane, including ceramic nanofiltration module/IL/silicon, showed much higher separation factor (selectivity) in comparison with ceramic nanofiltration module/IL and empty nanofiltartion module. Furthermore, the multiphase membrane which was coated by silicon displayed a good stability against ILs leaching. However, the permeation flux was decreased for multiphase membrane containing ceramic nanofiltration module/IL/silicon compared to empty ceramic nanofiltration module. Table 3 shows a summary report concerning ILs leaching and methods for its reducing.

## 5. Conclusions and Prospects

Today, PEMFC is recognized as a very promising source of sustainable energy. This kind of electrochemical device includes MEA, which is made by several parts, such as PEM, anode, and cathode catalyst layer; gas diffusion layer; bipolar plate; and gasket. Among all of them, PEM has a great potential to improve the efficiency of PEMFC. In the last decade, a wide range of modifications have been done on PEM in order to make them usable at middle and elevated temperatures under different humid conditions, such as using nanoparticles, sulfonated hydrocarbon polymers, and PA. These methods have their own limitations, including water evaporation, dehydration, and decreasing mechanical stability. The usage of ILs is a way to overcome these drawbacks since ILs are organic salts with negligible vapor pressure, excellent proton conductivity, and thermal stability. A wide range of ILs have been identified, including FILs possessing different ion exchange groups, such as sulfonate, sulfate, imide, and phosphate. The usage of FILs resulted in increasing the proton conductivity, thermal, and chemical stability of membranes in comparison with the pristine ones. However, membranes did suffer from leakage of ILs during the process. There are some techniques that allow us to reduce the ILs leakage, such as the application of hydrophobic ILs, inorganic compounds, inorganic NPs, and mesoporous fillers, as well as providing optimum operation condition. Despite researchers’ efforts and the number of articles published, currently, the usage of ionic liquids-based membranes for middle and high temperature-polymer electrolyte membrane fuel cells still remains limited. Nevertheless, promising findings have been reported. As a short-term future prospect, the use of composite membranes containing different types of ILs in various separation processes will be increased owing to tunable properties of ILs. As for long-term future prospects, it is considered that researchers are going to overcome leaching during the separation process which will lead to enhancement of the membrane operation life-time.

## Figures and Tables

**Table 1:** Different types of ILs and their characteristics.

Type of Ionic Liquid	Abbreviated Name	Summary and Property	Applications	Ref.
Chiral ionic liquids	C-ILs	The best option for asymmetric induction in catalysis. Because of the nature of C-ILs, the process of their synthesis is difficult (needed several steps) and expensive. These kinds of ILs are usually synthesized by asymmetric synthesis or chiral pool and they can contain polar, axial or central chirality.	Liquid chiral chromatography, stereo selective polymerization, synthesis of potential active chiral compounds, liquids crystal, NMR chiral discrimination, solvent, electrolyte, and catalyst.	[78]
Switchable polarity solvent ionic liquids	SPS-ILs	SPS-ILs are usually synthesized by proton transfer reaction. Additionally, SPS-ILs have acceptable resistance against wet condition for synthesis and operation. By adjusting the value for molecular triggers, including CO_2_ and CS_2_, the physical features of SPS-ILs can be changed.	Solvent recovery and solute separation.	[112]
Protic ionic liquids	Pr-ILs	Pr-ILs can be quickly synthesized by transferring hydrogen ion (H^+^) from a Brønsted acid to a Brønsted base. The process of proton-transfer is boosted by using strong bases or acids or both of them. These ILs have good proton conductivity, fluidity, and low melting point.	Alkaline batteries, fuel cells, dehydration, and choromatogeraphy (both liquid and gas).	[110]
Bio-ionic liquids	Bio-ILs	Bio-ILs are often produced by sustainable bio-precursors; therefore, they are environmentally friendly, bio-degradable, biocompatible, and non-toxic. They have high thermal (between 220 and 290ºC) stability and solubility (in methanol, Dimethyl sulfoxide, chloroform, and water).	Biodiesel production, renewable diesel and jet fuel, chemical compounds production (like herbicides).	[107]
Poly- ionic liquids	P-ILs	P-ILs are also known as polymerized ionic liquids. P-ILs refer to a subclass of polyelectrolytes that feature an ionic liquid (IL) species in each monomer repeating unit, connected through a polymeric backbone to form a macromolecular architecture. In spite of the high charge density of P-ILs, they usually have wide glass transition temperature ranges.	Polymer electrolytes, batteries, fuel cells, carbon electrodes, sensors, organic transistors, super capacitors, catalysts, photoresists, and corrosion inhibitors.	[106]
Energetic ionic liquids	E-ILs	E-ILs have low melting point, and high thermal stability and can be used as eco-friendly explosives. These ILs have very low vapor pressure and structural designability. Due to the great safety and energy, as well as low negative environmental impacts on the eco-system of E-ILs, they can be good alternative instead of energetic materials, such as HMX, RDX, TNT, and CL-20.	Explosives, pyrotechnics, and propellants.	[109]
Neutral ionic liquids	*N*-ILs	In these ILs, the electrostatic interactions between anions and cations are typically very weak. Moreover, *N*-ILs hold low melting point and viscosity. As a result, *N*-ILs are usually used as neutral solvents.	Solvent.	[78]
Metallic ionic liquids	M-ILs	These types of ILs contain metal halides (e.g., (AlCl_3_^−^), (CuCl_3_^−^), (SnCl_3_^−^), and (Al_2_Br_7_^−^)). M-ILs are highly viscous in comparison with other types of ILs. M-ILs are typically stable under moisture and ambient conditions.	Catalyst, solvent, organometallic chemistry hydration process, and recycling of nuclear waste.	[105]
Basic ionic liquids	B-ILs	B-ILs are regarded as eco-friendly, flexible, non-volatile, active and selective catalysts; thus, B-ILs are good alternatives for conventional bases (e.g., KOH, NaOH, and NaHCO_3_). Unlike traditional bases, B-ILs do not suffer from environmental issue, waste production, and corrosion.	Organic transformation (e.g., Michael addition, aldol condensation, Knoevenagel condensation, Henry reaction, oximation, and Michael reaction), catalyst, and solvent.	[111]
Supported ionic liquids	S-ILs	The use of S-ILs have been increased because of high cost of pure ILs utilization. These ILs are usually benefited from silica support; hence, the requirement for using ILs significantly reduced. The application of S-ILs can accelerate exploitation of ILs in industrial and commercial processes.	Solvent, catalyst, reactor systems, and separation process.	[108]

**Table 3:** A summary report with regard to ILs leakage and several techniques for its diminishing.

Membrane	Types of Investigations Concerning IL Leaching	Observations	Ref.
(EMIm)(DEP)/SPEEK	Influence of the IL content.	The results confirmed that the enhancement of both IL content and operation temperature resulted in increasing the proton conductivity, in which the maximum conductivity was 3.16·10^−3^ Scm^−1^ at 145 °C for membrane with 50 wt% IL. However, increasing the IL content showed opposite impact on the leaching of IL from the membranes, and the membrane sample with 10 wt% (EMIm)(DEP) demonstrated the least IL leaching. The order of leaching after 48 h immersion was: SPEEK/IL-50% = 28% > SPEEK/IL-30% = 19.11% > SPEEK/IL-10% = 9.20%. The main reason is that the large amount of IL cannot be well bonded with polymer body. Moreover, it is stated that the primary absorption of IL on the polymer matrix is important, and, at lower IL concentrations, this interaction is stronger.	[156]
(bmim)(OTf)/SPEK (bmim)(NTf_2_)/SPEK	Influence of the operation temperature, hydrophilicity or hydrophobicity nature, and content of ILs.	The results revealed the rising the temperature brought about increasing the leakage. In addition, IL with hydrophilic nature ((bmim)(OTf)) demonstrated more leakage from membrane than hydrophobic one ((bmim)(NTf_2_)) in that hydrophilic compounds easily wash with water due to their nature affinity. Moreover, enhancing the concentration of ILs caused increasing the leakage.	[157]
(EIm)(TFSI)/PVDF-co-HFP (MIm)(TFSI)/PVDF-co-HFP (MPy)(TFSI)/PVDF-co-HFP	Utilization of inorganic compounds (Al_2_O_3_ or SiO_2_) and various cations.	(MPy)(TFSI)/PVDF-co-HFP composite membrane (with 60 wt% (MPy)(TFSI)) showed the least IL leakage among all membranes containing ILs (the order of ILs leakage is: (EIm)(TFSI) > (MIm)(TFSI) > (MPy)(TFSI)). Basically, the main reason is that (MPy) cation is stronger base than two others. Therefore, this IL can be more stable and less affected by water. Furthermore, the results showed that the addition of inorganic compounds (Al_2_O_3_ or SiO_2_) could reduce the leakage of ILs from the samples. The composite membrane comprising (MPy)(TFSI)/Al_2_O_3_ showed a better retention ability of IL than (MPy)(TFSI)/SiO_2_ because Al_2_O_3_ demonstrated much absorbent features with organic compounds, thanks to the hydrogen bondings.	[158]
(dema)(OTf)/SiO_2_/SPEEK	Influence of the sulfonation degree and silica addition of silica.	The results illustrated that, by increasing the sulfonation degree, IL leaching were decreased from the membranes. The main reason is that the existence of electrostatic interaction between ILs cation and sulfonic groups (on the structure of SPEEK) can diminish the leaching of ILs from the membranes. Nonetheless, further enhancement of sulfonation degree (more than 83%) led to increasing the leakage of ILs from the membrane owing to rising the hydrophilicity. Besides, adding silica decreased the leaching because this inorganic compound holds reactive sites which have great ability to interact with IL.	[144]
(EIm)(TfO)/(TMI)(Cl)-silica NPs/polymerizable oil	Using SiO_2_ NPs.	Due to the fact that SiO_2_ NPs are nanoscaled, they can be easily dispersed into the membrane and could react with IL; hence, the composite samples comprising NPs indicated much better retention ability than pristine ones. The results showed that the membranes without SiO_2_ NPs dramatically lost ILs (almost 90 wt%) after 10 min immersion in water, while the sample with 1 wt% SiO_2_ NPs demonstrated better result after 10 min (approximately 70 wt% IL weight loss).	[159]
(EIm)(TfO)/mesoporous silica/SPEEK (dema)(TfO)/mesoporous silica/SPEEK (BMIm)(TfO)/mesoporous silica/SPEEK (BMIm)(Cl)/mesoporous silica/SPEEK (BMIm)(BF_4_)/mesoporous silica/SPEEK	Influence of mesoporous silica and different types of IL cations.	Employing porous silica decreased the weight loss of ILs because not only does silica hold a number of reactive sites in its structure, but it also provides large pores which can trap ILs. Additionally, among three ILs with the same anion ((TfO)), the order of leaching was: (dema)(Tfo) > (EIm)(Tfo) > (BMIm)(Tfo) and the minimum IL weight loss was for the hybrid membrane composed of (BMIm) cation, in which this result revealed that the type of cation has a direct influence on leaching.	[160]
PAMAM G4.0-NH_3_^+^H_2_PO_4_^−^ PAMAM G4.0-NH_3_^+^HSO_4_^−^ PAMAM G4.0-NH_3_^+^Tf_2_N^−^	Influence of the nature of ILs.	The hydrophilic ILs-based membranes ((PAMAM G4.0-NH_3_^+^HSO_4_^−^)) > PAMAM G4.0-NH_3_^+^H_2_PO_4_^−^)) showed a better conductivity, whereas the hydrophobic one ((PAMAM G4.0-NH_3_^+^Tf_2_N^−^)) had better stability with regard to leakage phenomenon. The composite membrane possessing (PAMAM G4.0-NH_3_^+^Tf_2_N^−^) lost only 35 wt% of IL after 120 min, while the membrane containing (PAMAM G4.0-NH_3_^+^H_2_PO_4_^−^) showed worse IL leaching in the same condition (around 90 wt%).	[155]
Ceramic nanofiltration module/((C_3_H_7_)_4_N)(B(CN)_4_))/silicon	Influence of coating silicon.	The multiphase membrane containing ceramic nanofiltration module/((C_3_H_7_)_4_N)(B(CN)_4_)) was coated by silicon showed the highest seperation factor of 177. Moreover, coating the silicon led to increasing the stability of ILs in the structure of membrane (more than 9 months). The modified membrane showed low permeation flux (3.86 g/m^2^h).	[161]

## Data Availability

Not applicable.

## Supplementary Materials

The following are available online at https://www.mdpi.com/article/10.3390/ijms22115430/s1, Table S1: The abbreviation and full chemical name of some common ILs.

## Abbreviations

AFC	Alkaline fuel cell
A-ILs	Aprotic ILs
B-ILs	Basic ionic liquids
Bio-ILs	Bio-ionic liquids
C-ILs	Chiral ionic liquids
CO	Carbon monoxide
CODR	Chemical organic demand removal
DMFC	Direct methanol fuel cell
E-ILs	Energetic ionic liquids
FIL	Functionalized ionic liquid
GO	Graphene oxide
HFP	Hexafluoropropylene
HT-PEMFC	High temperature proton exchange membrane fuel cell
IEC	Ion exchange capacity
ILC	Ionic liquid cation
ILs	Ionic liquids
LT-PEMFC	Low temperature polymer electrolyte membrane fuel cell
MCFC	Molten carbonate fuel cell
MEA	Membrane electrode assembly
MFC	Microbial fuel cell
M-ILs	Metallic ionic liquids
MT-PEMFC	Middle temperature proton exchange membrane fuel cell
*N*-Ils	Neutral ionic liquids
NPs	Nanoparticles
OCP	Open circuit potential
PA	Phosphoric acid
PAFC	Phosphoric acid fuel cell
PAMAM	Polyamidoamine
PBI	Polybenzimidazole
PEM	Polymer electrolyte membrane
PEMFC	Proton exchange membrane fuel cell
PFSA	Perfluorosulfonic acid
PI Polyimide	
P-Ils	Poly-ionic liquids
Pr-Ils	Protic ionic liquids
PVC	Polyvinylchloride
PVDF	Polyvinyldenefluoride
QAPSU	Quaternary ammonium functionalized polysulfone
QPSU	Quaternary polysulfone
RH	Relative humidity
RT-ILs	Room temperature ionic liquids
SAN Poly	(styrene-co-acrylonitrile)
SBA-15	Santata Barbara amorphous-15
SILMs	Supported ionic liquid membranes
S-ILs	Supported ionic liquids
SOFC	Solid oxide fuel cell
SPEEK	Sulfonated poly (ether ether) ketone
SPEK	Sulfonated poly (ether ketone)
SPI	Sulfonated polyimide
SPS-ILs	Switchable polarity solvent-ILs
TnT	Titanate nanotubes
TS-ILs	Task specific-ionic liquids
