# Supplementary material for: A Review on Ionic Liquids-Based Membranes for Middle and High Temperature Polymer Electrolyte Membrane Fuel Cells (PEM FCs)
Source: Int J Mol Sci. 2021 May 21;22(11):5430. doi: 10.3390/ijms22115430 (PMC8196583; doi:10.3390/ijms22115430)
Supplement: Supplementary file 1 [file ijms-22-05430-s001.zip › ijms-1224556-supplementary.pdf]

Table S1. The abbreviation and full chemical name of some common ILs.

Abbreviation	Full chemical name
[A336][C272]	[Trialkylmethylammonium][bis 2,4,4-trimethyl-pentylphosphinate]
[A336][Cl]; Aliquat 336	Tricaprylylmethylammonium chloride
[A336][DEHP]	Tri-n-octylmethylammonium bis(2-ethylhexyl)phosphate
[A336][DGA]	Tri-noctylmethylammonium bis(2-ethylhexyl)diglycolamate
[A336][MTBA]	Tricaprylylmethylammonium 2-(methylthio)benzoate
[bmim][PF$_6$]	1-butyl-3-methylimidazolium hexafluorophosphate
[bmim] [BF$_4$]	1-butyl-3-methylimidazolium tetrafluoroborate
[bmim][NO$_3$]	1-butyl-3-methylimidazolium nitrate
[bmim][Tf$_2$N]	1-butyl-3-methylimidazolium bis(trifluoromethylsulfonyl)imide
[bmim][TfO]	1-butyl-3-methylimidazolium trifluoromethanesulfonate
[bmim][SCN]	1-butyl-3-methylimidazolium thiocynate
[bmim][DCA]	1-butyl-3-methylimidazolium dicyanamide
[bmim][Ac]	1-butyl-3-methylimidazolium acetate
[bmim][CH$_3$SO$_3$]	1-Butyl-3-methylimidazolium methanesulfonate
[bmim][TFSI]	1-Butyl-3-methylimidazolium bis(trifluoromethanesulfonyl) imide
[bmim][Br]	1-Butyl-3-methylimidazolium bromide
[bmim][Cl]	1-Butyl-3-methylimidazolium chloride
[bmim][FeCl$_4$]	1-Butyl-3-methylimidazolium tetrachloroferrate
[bbim][BF$_4$]	1-Benzyl-3-butylimidazolium tetrafluoroborate
[BMP][Tf$_2$N]	1-butyl-1-methylpyrrolidinium bis(trifluoromethylsulfonyl)imide
[C$_4$mim][Tf$_2$N]	1-butyl-3-methylimidazolium bis(trifluoromethyl)sulfonylamide
[C$_6$mim][PF$_6$]	1-hexyl-3-methylimidazolium hexafluorophosphate
[C$_8$mim][PF$_6$]	1-octyl-3-methylimidazolium hexafluorophosphate
[C$_9$mim][PF$_6$]	1-nonyl-3-methylimidazolium hexafluorophosphate
[C$_6$mim] [BF$_4$]	1-hexyl-3-methylimidazolium tetrafluoroborate
[C$_8$mim][BF$_4$]	1-octyl-3-methylimidazolium tetrafluoroborate
[C$_{10}$mim][Tf$_2$N]	1-decyl-3-methylimidazolium bis(trifluoromethyl)sulfonylamide
Cyanex 272	Bis(2,4,4-trimethylpentyl)phosphinic acid
Cyanex 302	Bis(2,4,4-trimethylpentyl)monothiophosphinic acid
[dmim][Tf$_2$N]	1,2-dimethylimidazolium bis(trifluoromethylsulfonyl)imide
[dema][Tf$_2$N]	Diethylmethylammonium bis(trifluoromethane sulfonyl)amide
[dema][TfO]	Diethylmethylammonium trifluoromethanesulfonate
D$_2$EHPA	Di(2-ethylhexyl)phosphoric acid
[emim][BF$_4$]	1-ethyl-3-methylimidazolium tetrafluoroborate
[emim][Tf$_2$N]	1-ethyl-3-methylimidazolium bis(trifluoromethylsulfonyl)imide
[emim][pivalate]	1-ethyl-3-methylimidazolium pivalate
[emim][C$_2$N$_3$]	1-ethyl-3-methylimidazolium dicyanamide
[emim][Ac]	1-ethyl-3-methylimidazolium acetate
[emim][MDEGSO4]	1-ethyl-3-methylimidazolium 2-(2-methoxyethoxy)ethylsulfate
[emim][lactate]	1-ethyl-3-methylimidazolium lactate
[emim][benzoate]	1-ethyl-3-methylimidazolium benzoate
[HJMT][Cy272]	Tertiary alkyl (C6-C12) primary ammonium bis 2,4,4-(trimethylpentyl)phsphinate
HEF	2-hydroxy ethylammonium formate
HEAF	2-(2-hydroxy ethoxy)-ammonium formate
HEAA	2-(2-hydroxy ethoxy)-ammonium acetate
[HOPmim][NO$_3$]	Hydroxypropylmethylimidazolium nitrate
[MPS$_2$PIP][Tf$_2$N]	1-methyl-1-[4,5-bis(methylsulfide)]pentylpiperidi-nium bis(trifluoromethylsulfonyl)imide
	1-methyl-2-pentenepyrrolidinium bis(trifluoromethylsulfonyl)imide
[MPTPYRRO][Tf$_2$N]	1-methyl-1-octylpiperidinium bis(trifluoromethylsulfonyl)imide
[MOPIP][Tf$_2$N]	9-methyl-2,3,4,6,7,8-hexahydropyrimido[1,2-a]pyrimidine phenol
[MTBDH][PhO]	9-methyl-2,3,4,6,7,8-hexahydropyrimido[1,2-a]pyrimidine (1- phenyl)trifluoroethanol
[MTBDH][TFPA]	9-methyl-2,3,4,6,7,8-hexahydropyrimido[1,2-a]pyrimidine imidazole

[MTBDH][Im]	
[MTBDH][TFE]	9-methyl-2,3,4,6,7,8-hexahydropyrimido[1,2-a]pyrimidine trifluoroethanol
[MTOA][Cl]	Methyl trioctyl ammonium chloride
$[N_{4444}]$[DEHP]	Tetrabutylammonium bis (2-ethylhexyl)phosphate
[N-bupy]$[BF_4]$	N-butylpyridinium tetrafluoroborate
$[NH_2$p-bim]$[BF_4]$	1-propylamide-3-butyl imidazolium tetrafluoroborate
$[NBu_4]$[(PFOc)SO_3]	Tetrabutylammonium heptadecafluorooctanesulfonate
$[N_{1111}]$[Gly]	Tetramethylammonium glycinate
[OMIM]$[BF_4]$	1-octyl-3-methylimidazolium tetrafluoroborate
[OMIM]$[Tf_2N]$	1-octyl-3-methylimidazolium bis(trifluoromethylsulfonyl)imide
[OMIM]$[PF_6]$	1-Octyl-3-methylimidazolium hexafluorophosphate
[OMIM]$[FeCl_4]$	1-Octyl-3-methylimidazolium tetrachloroferrate
$[P_{6\ 6\ 6\ 14}]$$[Tf_2N]$	Trihexyltetradecylphosphonium bis(trifluoromethyl)sulfonylamide
$[P_{14,6,6,6}]$$[Tf_2N]$	Trihexyltetradecylphosphonium bis(trifluoromethylsulfonyl)imide
$[P(C_4)_4]$[Ala]	Tetrabutylphosphonium l-α-aminopropionic acid salt
$[P(C_4)_4]$[Gly]	Tetrabutylphosphonium aminoethanoic acid salt
$[P_{66614}]$[Met]	Trihexyl(tetradecyl)phosphonium methioninate
$[P_{66614}]$[Pro]	Trihexyl(tetradecyl)phosphonium prolinate
$[(P_2$-Et)H][TFE]	Tetramethyl(tris(dimethylamino)phosphoranylidene)phosphorictriamid-Et-imin trifluoroethanol
$[(P_2$-Et) H][Im]	Tetramethyl(tris(dimethylamino)phosphoranylidene)phosphorictriamid-Et-imin imidazole
$[(P_2$-Et) H][Pyrr]	Tetramethyl(tris(dimethylamino)phosphoranylidene)phosphorictriamid-Et-imin pyrrolidone
$[(P_2$-Et) H][PhO]	Tetramethyl(tris(dimethylamino)phosphoranylidene)phosphorictriamid-Et-imin phenol
$[P_{66614}]$[Pyr]	Trihexyl(tetradecyl)phosphonium pyrazole
$[P_{66614}]$[Im]	Trihexyl(tetradecyl)phosphonium imidazole
$[P_{66614}]$[Ind]	Trihexyl(tetradecyl)phosphonium indole
$[P_{66614}]$[Triz]	Trihexyl(tetradecyl)phosphonium Trizole
$[P_{66614}]$[Bentriz]	Trihexyl(tetradecyl)phosphonium bentrizole
$[P_{66614}]$[Tetz]	Trihexyl(tetradecyl)phosphonium tetrazole
$[P_{66614}]$[Oxa]	Trihexyl(tetradecyl)phosphonium oxazolidinone
$[P_{66614}]$[PhO]	Trihexyl(tetradecyl)phosphonium phenol
$[P_{6,6,6,14}]$$[GdCl_6]$	Trihexyl(tetradecyl)phosphonium hexachlorogadolinium
$[P_{6,6,6,14}]$$[CoCl_4]$	Trihexyl(tetradecyl)phosphonium tretrachlorocobalt
$[P_{6,6,6,14}]$$[FeCl_4]$	Trihexyl(tetradecyl)phosphonium tretrachloroferrate
$[P_{6,6,6,14}]$$[MnCl_4]$	Trihexyl(tetradecyl)phosphonium tretrachloromanganese
poly[META][Cl]	Poly([2-(methylacryloyloxy)ethyl]trimethylammonium) chloride
poly[SMIM]$[Tf_2N]$	Poly(1-styrenemethyl-3-methylimidazolium bis(trifluoromethylsulfonyl)imide)
poly[ViEtIm]$[Tf_2N]$	Poly(1-vinyl-3-ethyl-imidazolium) bis(trifluoromethane sulfonyl)amide
poly[ViEtIm][Br]	Poly(1-vinyl-3-ethyl-imidazolium) bromide
poly[ViEtIm]$[BF_4]$	Poly(1-vinyl-3-ethyl-imidazolium) tetrafluoroborate
pr$[MIM]_2[Tf_2N]_2$	1,3-Di(3-methylimidazolium)propane bis(trifluoromethylsulfonyl)imide
[QP][Cl]; Cyphos IL 101	Trihexyl(tetradecyl)phosphonium chloride
[QP][Br]; Cyphos IL 102	Trihexyl(tetradecyl)phosphonium bromide
[SPMIM]$[PF_6]$	1-(Silylpropyl)-3-methyl-imidazolium hexafluorophosphate
THEAA	Tri-(2-hydroxy ethyl)-ammonium acetate
TEGO IL K_5	Quaternary ammonium compounds, coco alkylbis (hydroxyethyl)methyl, ethoxylated, chlorides, methyl chloride
